# Short-term power load forecasting in China: A Bi-SATCN neural network model based on VMD-SE

**DOI:** 10.1371/journal.pone.0311194

**Published:** 2024-09-30

**Authors:** Yuan Huang, Qimeng Feng, Feilong Han

**Affiliations:** School of Information and Electrical Engineering, Hebei University of Engineering, Handan, Hebei, China; Univerzitet Singidunum, SERBIA

## Abstract

This study focuses on improving short-term power load forecasting, a critical aspect of power system planning, control, and operation, especially within the context of China’s "dual-carbon" policy. The integration of renewable energy under this policy has introduced complexities such as nonlinearity and instability. To enhance forecasting accuracy, the VMD-SE-BiSATCN prediction model is proposed. This model improves computational efficiency and reduces prediction errors by analyzing and reconstructing sequence component complexity using sample entropy (SE) following variational mode decomposition (VMD). Additionally, a self-attention mechanism is integrated into the temporal convolutional network (TCN) to overcome the traditional TCN’s limitations in capturing long-term dependencies. The model was evaluated using data from the China Ninth Electrical Attribute Modeling Competition and validated with real-world data from a specific county in Shijiazhuang City, Hebei Province, China. Results indicate that the VMD-SE-BiSATCN model outperforms other models, achieving a mean absolute error (MAE) of 92.87, a root mean square error (RMSE) of 126.906, and a mean absolute percentage error (MAPE) of 0.81%.

## 1. Introduction

As large quantities of power resources cannot be retained as instantaneous energy sources, the generation and consumption of electrical loads must occur simultaneously. Relying on historical data, short-term power load forecasting reflects the trends in future power consumption. By leveraging power forecasting results, the power generation department can develop production and scheduling plans, thereby significantly enhancing the efficiency of the power system. The aspiration and objective of "striving to peak carbon dioxide emissions by 2030 and achieve carbon neutrality by 2060" was declared by the Chinese government in recent years [1]. The incorporation of new energy sources leads to a more varied energy structure, which accentuates the nonlinearity and instability of loads. As a result, load forecasting faces increasingly complex challenges. Precise prediction plays a crucial role in ensuring the secure and stable operation, as well as the advancement, of the power system [2].

Short-term load forecasting is an essential foundation for power system scheduling and planning, necessitating the creation of increasingly accurate prediction models. Current models for short-term load forecasting can be broadly categorized into two groups: traditional methods [3,4] and artificial intelligence methods [5–7]. Common traditional techniques include regression analysis [8] and Kalman filtering [9]. However, conventional techniques are typically constrained by their frameworks and often perform poorly when handling complex nonlinear time-series data. With advancements in high-performance computing and big data technology, artificial intelligence methods have become the primary choice for prediction, resulting in significant improvements in modeling nonlinear temporal characteristics. Artificial intelligence techniques encompass machine learning [10–13] and deep learning [14–17], with deep learning techniques dominating the field. Presently, the prevailing approaches for short-term power load forecasting primarily are based on recurrent neural network (RNN) models [18]. Long short-term memory (LSTM) neural networks and Gated Recurrent Unit Network (GRU) are the most representative, as they effectively mitigate the gradient vanishing problem common in conventional RNNs during long-term sequences. Kwon et al [19] use the LSTM model to estimate power load time series data through regression, considering the impact of various temporal dimensions on the load. In their study, Yan et al [20] employed the Bi-LSTM model as a power load prediction model to enhance the accuracy of forecasting results. Some researchers have integrated the feature extraction capabilities of convolutional neural networks (CNN) into hybrid prediction models, conducting extensive research in this area. Wu et al [21] proposed a short-term power load forecasting model based on CNN and BiLSTM, where CNN effectively extracts features, while the LSTM-BiLSTM layers predict the load based on these extracted features.

Although the extraction capability can be improved by stacking CNN layers, the receptive field of deep CNN layers is limited by the fixed convolution kernel size. The temporal convolutional network (TCN) model [22–24] can effectively extract features from complex nonlinear time series and generate predictions. Its dilated causal convolutional component significantly improves the learning capability of deep networks. Wang et al [25] used the TCN model for power load forecasting. TCN offers the advantage of supporting massively parallel processing, unlike RNN forecasting. However, traditional TCNs still face the problem of limited receptive fields. Some researchers have incorporated an attention mechanism into the network architecture to improve the model’s performance, enabling it to extract more relevant information features. Cheng and colleagues [26] incorporated the Convolutional Block Attention Module (CBAM), which combines spatial and channel attention mechanisms, into a TCN model. The application of TCN-CBAM in predicting complex chaotic time series demonstrated its ability to extract time series features more efficiently. Tang et al [27] combined TCN with a temporal attention mechanism, effectively extracting the nonlinear relationship between meteorological factors and load. Tong et al [28] proposed an Attention-based Spatiotemporal Convolutional Network (ACN), which integrates a one-dimensional convolutional block attention module (CBAM) structure, achieving the highest prediction accuracy with fewer parameters.

Extracting beneficial trends from power load series data is challenging for a single forecasting model due to the nonlinear, variable, and stochastic nature of the data. Analyzing valuable data from power load series that exhibit significant fluctuations remains a major challenge. Time series decomposition techniques, such as wavelet analysis (WA) [29,30] and empirical mode decomposition (EMD) [31–33], have been widely employed by researchers in power load forecasting. Meng et al. [34] developed a prediction model integrating EMD with Bi-LSTM. However, this approach is susceptible to modal aliasing when sudden changes occur in the signal. As a result, more researchers are turning to variational mode decomposition (VMD) [35,36], which can dynamically determine the number of decompositions based on specific conditions. This method offers greater adaptability and effectively reduces the likelihood of modal aliasing. To further enhance prediction accuracy, Ran et al [37] employed sample entropy (SE) to recombine the data after mode decomposition, which enhances the efficiency of feature extraction. Table 1 summarizes the applications of machine learning and deep learning in power load forecasting. Therefore, the short-term power load forecasting model presented in this research employs a hybrid decomposition technique combining VMD and SE to effectively dissect load sequences and extract their characteristics.

**Table 1:** Summary of uses of machine learning and deep learning schemes in power load forecasting.

Method	Comparative methods	MAPE (%)	RMSE	References
SVM-RF-LSTM	SVM, RF, LSTM	0.0285	10.0746	[10]
SVR-CDSES	SVR, BPNN, LR, RF	7.20	\	[13]
1-D CNN	LSTM, GRU	1.0002	\	[14]
CNN, Bidirectional LSTM-GRU	FFNN, LSTM, GRU,	0.68	\	[17]
GRU	ARIMA	\	8.1331	[18]
LSTM	KSLF	1.39	\	[19]
CNN-LSTM-BiLSTM	SVR, RFR, BPNN,	\	6.396	[21]
TCN-CBAM	LSTM, CNN-LSTM, TCN	\	2.3803	[26]
TCN-MIC-Attention	LSSVM, SVR, BPNN, TCN, LSTM	4.57	447.45	[27]
EMD-Bi-LSTM	EMD-Bi-GRU	0.28	0.31	[33]
VMD-tsPSO-LSSVR	SVR, ANN, VMD-LSSVR, EMD-LSSVR	0.0077	245.544	[35]
CEEMDAN-SE-TR	LSTM, TR, GP, EMD-SE-TR	4.80	1.26	[37]

**RF**, random forest; **SVR-CDSES**, support vector regression-compositional data second exponential smoothing; **LR**, logistic regression; **BPNN**, backpropagation neural network; **FFNN**, feed forward neural network; **KSLF**, KPX short-term load forecasting; **RFR**, random forest regression; **MIC**, maximum information coefficient; **LSSVM**, least square support vector machine; **TR**, Transformer; **GP**, gauss process regression.

In conclusion, this research proposes a bi-directional SATCN model for predicting power load data, with initial decomposition performed using VMD technology. To facilitate feature extraction for load sequences with high fluctuations, SE is also employed to reconstruct the sequences following modal decomposition. The experimental data for this study were obtained from the regional load dataset of the China Ninth Electrical Attribute Modeling Competition. The validation data were collected from a county in Shijiazhuang City, Hebei Province, China.

The following are the contributions of this study:

1. The study develops a predictive model rooted in the principles of deconstruction and reconstruction. Most contemporary research employs modal decomposition of time series and subsequently inputs the decomposed individual component series directly into forecasting models. These forecasting approaches tend to ignore the interdependence among the decomposed series, which leads to a higher margin of error. This study proposes the use of sample entropy to reconstruct the time series data following VMD decomposition, resulting in enhanced prediction accuracy and decreased model training complexity.
2. By incorporating a self-attention mechanism module into the TCN, it becomes possible to capture intrinsic sequence correlations and focus the network’s attention on the most relevant features.
3. The construction of a bidirectional SATCN model enables feature extraction from both forward and backward directions, thereby improving load forecasting capability.

The other sections of the paper are organized as follows: section 2 describes the methodology, section 3 details the presented model, section 4 analyzes and discusses the results of the experiment, and section 5 concludes the research.

## 2. Theories and methods

### 2.1 Variational Mode Decomposition (VMD)

Proposed in 2014, variational mode decomposition (VMD) is a fully non-recursive and adaptive technique for signal sequence decomposition. It can decompose a complex original signal into several simple intrinsic mode functions (IMFs) [38]. This approach effectively mitigates the endpoint effect and modal aliasing problems associated with empirical mode decomposition (EMD). As a result, VMD can process nonlinear and non-smooth signals more effectively. Because weather and seasonality can affect short-term power load, VMD is better equipped to adjust to these fluctuations, making it particularly well-suited for short-term power load forecasting.

Let’s assume that the original sequence *f*(*t*) is decomposed into *k* eigenmode functions *u*_*k*_(*t*):

$$u_{k}(t)=A_{k}(t)\;\cos\left(\omega_{k}(t)\right)$$ (1)

where *A*_*k*_(*t*) is the instantaneous amplitude and *A*_*k*_(*t*)≥0; *ω*_*k*_(*t*) is the instantaneous frequency.

**Fig 1:**
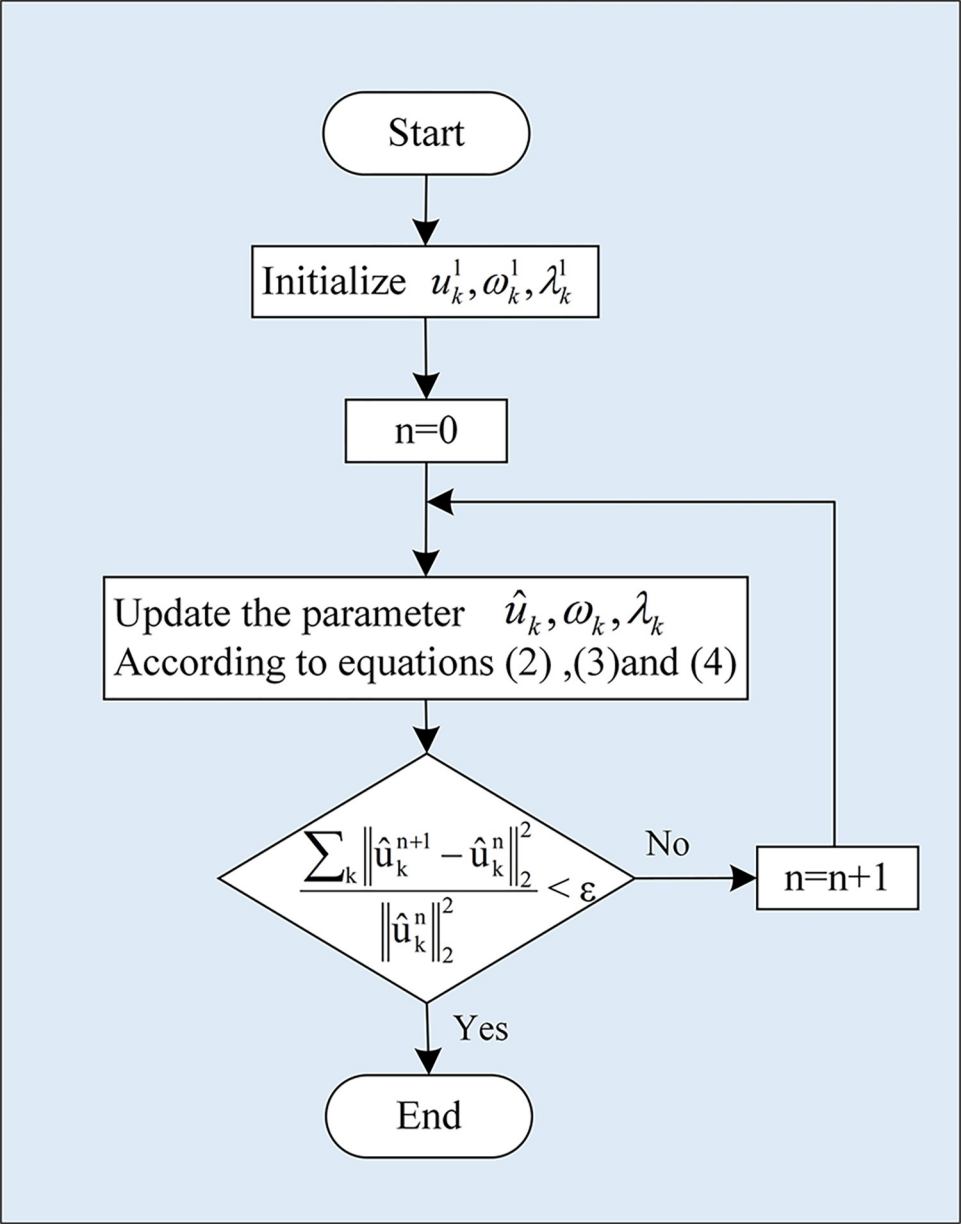
VMD flowchart.

The flow of VMD decomposition is shown in Fig 1 with the following steps:

1. Introduce a quadratic penalty factor *α* and the Lagrange multiplication operator *λ*(*t*) to construct an unconstrained variational problem. Set the initial values of $u_{k}^{1},\omega_{k}^{1}$, and *λ*^1^.
2. By alternately updating the values of $u_{k}^{n+1},\omega_{k}^{n+1}$, and *λ*^*n*+1^ to calculate the extreme points of the extended Lagrangian expression, and ultimately to determine the optimal set of load decomposition modes. The updated equation:
$${\hat{u}}_{k}^{n+1}(\omega )=\frac{\hat{f}(\omega )-\sum_{i\neq k}{\hat{u}}_{i}^{n+1}(\omega )+{\hat{\lambda }}^{n}(\omega )/2}{1+2\alpha {\left(\omega -\omega_{k}^{n}\right)}^{2}}$$ (2)
$$\omega_{k}^{n+1}=\frac{\int_{0}^{\infty }\omega {|{\hat{u}}_{k}^{n+1}(\omega )|}^{2}d\omega }{\int_{0}^{\infty }{|{\hat{u}}_{k}^{n+1}(\omega )|}^{2}d\omega }$$ (3)
$${\hat{\lambda }}^{n+1}(\omega )={\hat{\lambda }}^{n}(\omega )+\tau (\hat{f}(\omega )-\sum_{k}{\hat{u}}_{k}^{n+1}(\omega ))$$ (4)
  where *n* is the number of iterations; *τ* is the convex function optimization parameter.
3. The IMF component of a VMD decomposed original power load sequence is derived by iterative computation until either the convergence requirement is met. The overall flow of VMD decomposition is shown in Fig 1.

### 2.2 Sample Entropy (SE)

Based on approximate entropy (AE), sample entropy (SE) provides a numerical measure of time series complexity [39]. Compared to AE, SE calculates the approximation without involving data segment comparison and is independent of data length, offering better consistency and reducing the errors associated with AE.

After decomposing a time series, the resulting component sequences capture different frequency and amplitude components of the power load signal. These component sequences might share certain similarities. However, predicting each component sequence individually would escalate computational complexity and potentially amplify prediction errors. By reconstructing IMF component sequences using sample entropy, it becomes possible to more accurately and reliably depict the overall dynamic behavior of power load signals, thereby enhancing the precision and resilience of short-term power load forecasting. The specific steps for this calculation are outlined below:

Let the original data be {*u*(*i*),*i* =1,…,*N*} with *N* points.

1. Transform the sequence into an *m*-dimensional vector, i.e., *u*(*i*) = [*u*(*i*),*u*(*i*+1),…,*u*(*i*+*m*−1)], *i* = 1,2,…,*N*−*m*+1. Define *d*[*u*(*i*),*u*(*j*)] as the maximum difference of the corresponding elements between two vectors *u*(*i*) and *u*(*j*).
$$d[u(i),u(j)]=\underset{a=0∼m-1}{\max}\left\{u(i+a)-u(j+a)\right\}$$ (5)
  where *i*,*j*∈[1,*N*−*m*+1],*i*≠*j*.
2. Calculate the total number of *d*[*u*(*i*),*u*(*j*)]<*r* for each *u*(*i*) and determine the percentage share.
$$B_{i}^{m}(r)=1/(N-m+1)*num\{d[u(i),u(j)]<r\}$$ (6)
3. Compute the average of $B_{i}^{m}(r)$ for all *i*, denoted *B*^*m*^(*r*):
$$B^{m}(r)=1/(N-m+1)*\sum_{i=1}^{N-m+1}B_{i}^{m}(r)$$ (7)
4. Repeat steps (1) ~ (3) as the dimension *m* increases by 1 and compute *B*^*m*+1^(*r*).
5. The SampEn value of the final time series is expressed as:
$$SampEn(m,r)=-\ln\left(B^{m+1}(r)/B^{m}(r)\right)$$ (8)
  where the similarity tolerance *r* is usually 0.1 to 0.25 times the time series standard deviation, and the parameter dimension *m* is typically selected as 1 or 2.

### 2.3 Temporal Convolutional Network (TCN)

The temporal convolutional network (TCN) is a temporal model derived from convolutional neural networks (CNNs). The main architecture of TCN primarily consists of residual blocks and dilated causal convolutions.

TCN has proven effective in short-term power load forecasting. It can effectively capture dependencies in time series data, enabling a deeper understanding of power load fluctuation patterns. Furthermore, the parallel processing capability of TCN allows for efficient processing of large datasets, while its residual module architecture mitigates the issue of gradient vanishing and enhances training stability. This is particularly crucial when handling complex time series data, such as power load.

#### 2.3.1 Dilated Causal Convolution (DCC)

**Fig 2:**
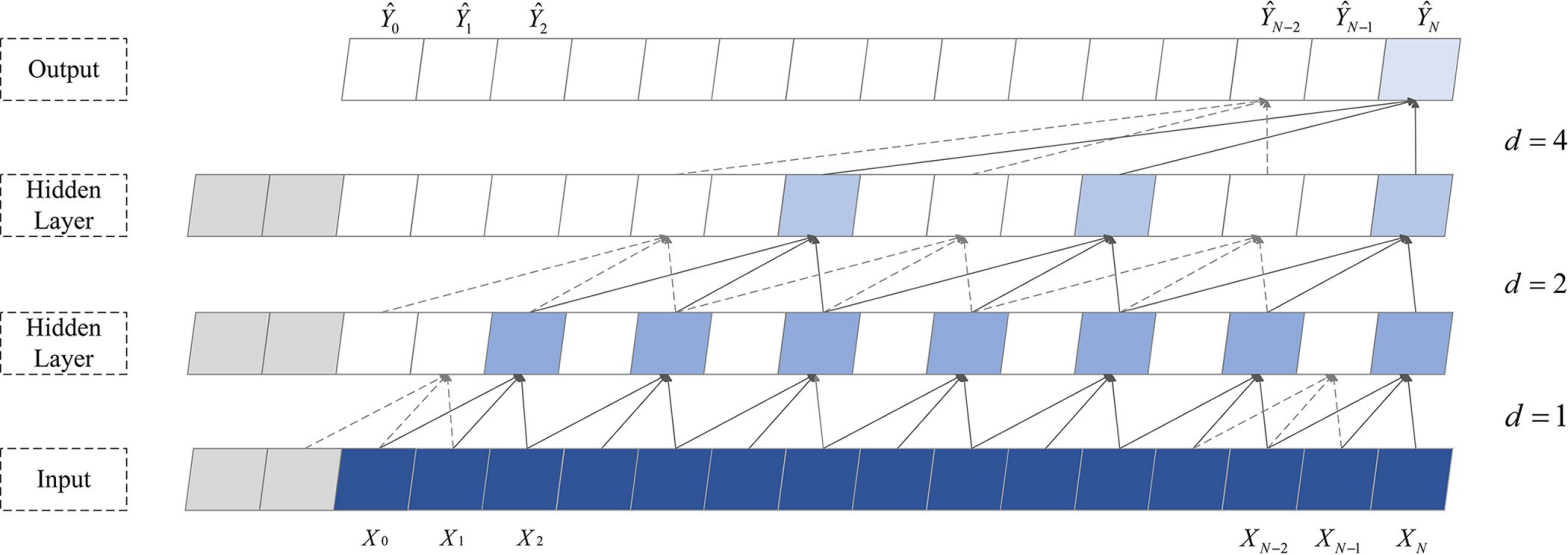
The architecture of dilated causal convolution with a convolution kernel size of k = 3.

Temporal convolutional networks utilize dilated causal convolution (DCC) to expand the receptive field, as depicted in Fig 2. DCC samples the input data at intervals, where *d* denotes the size of the interval. This approach allows for a larger receptive field to be achieved with fewer convolutional layers. The expression for dilated convolution is given by Eq (9).

$$F(t)=\sum_{i=0}^{k-1}h(i)X(t-d\cdot i)$$ (9)

where *X* represents the input data sequence and $\hat{Y}$ denotes the output results; *F*(*t*) represents the convolution result for the *t*-th element in the input data (*X*_0_,…,*X*_*t*_); *h*(*i*) is the *i*-th element in the convolution kernel; *k* represents the convolution kernel size, and *d* is the dilation factor.

#### 2.3.2 Residual Block (RB)

**Fig 3:**
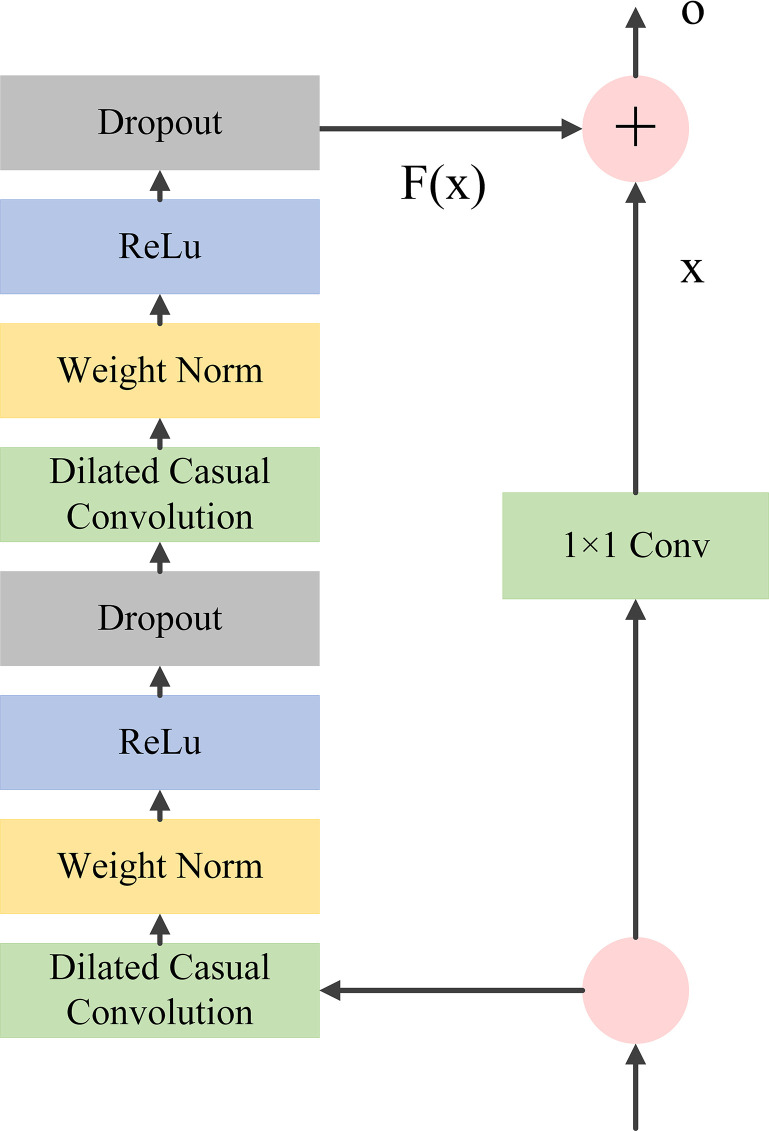
The architecture of residual block.

The residual block (RB) is a proven technique for overcoming the challenges associated with training deep networks. The architecture of the RB is illustrated in Fig 3. In TCN, the input to the residual block is denoted by *X*, and the output is represented by *o*, as shown in Eq (10).

O=Activation(X+F(X)) (10)

where *Activation* is the activation function, which in this research is set to ReLU.

The TCN network is constructed by stacking multiple residual blocks, each of which has two dilated causal convolution layers. The weight normalization layer (Weight Norm) standardizes the weights and normalizes the inputs to the hidden layers. The activation function, ReLU, introduces nonlinearities into the TCN network. Dropout regularization prevents overfitting, while residual connections directly map the inputs and mitigate network degradation caused by adding more layers.

### 2.4 Self-Attention mechanism (SA)

**Fig 4:**
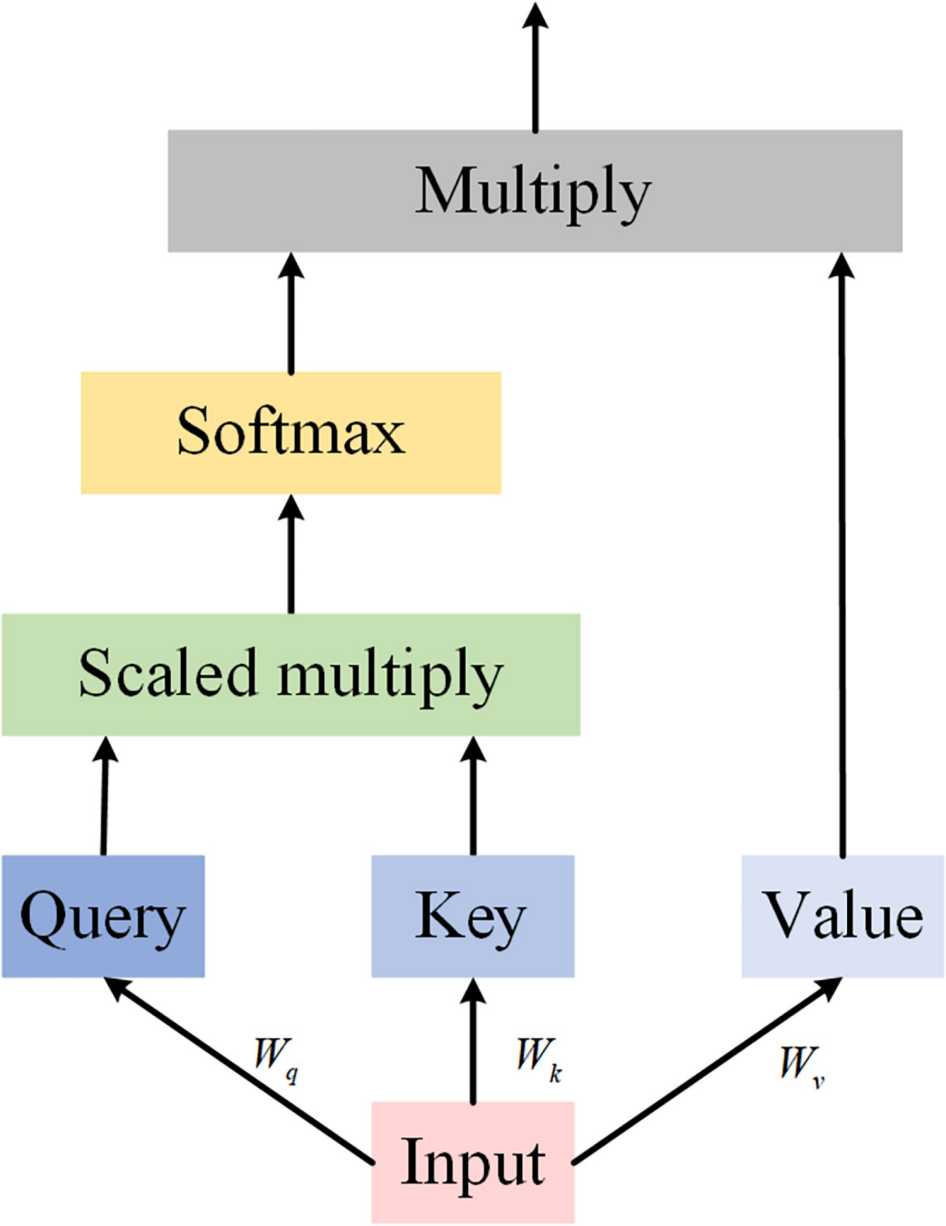
The architecture of the self-attention mechanism.

Vaswani et al originally proposed the self-attention mechanism [40], which allows for focusing on significant information while reducing attention to irrelevant data through weight allocation. The self-attention mechanism is specifically designed to capture interdependencies within a sequence. Fig 4 illustrates the self-attention architecture, where the input data is multiplied by three weight matrices—*W*_*q*_,*W*_*k*_, and *W*_*v*_, to produce three vectors: *Q* (query), *K* (key), and *V* (value).

The specific arithmetic is shown below:

$$Attention(Q,K,V)=softmax(QK^{T}/\sqrt{d_{K}})V$$ (11)

where *d*_*K*_ is the dimension of *Q*,*K* and $\sqrt{d_{K}}$ plays a moderating role.

## 3. The proposed hybrid model

**Fig 5:**
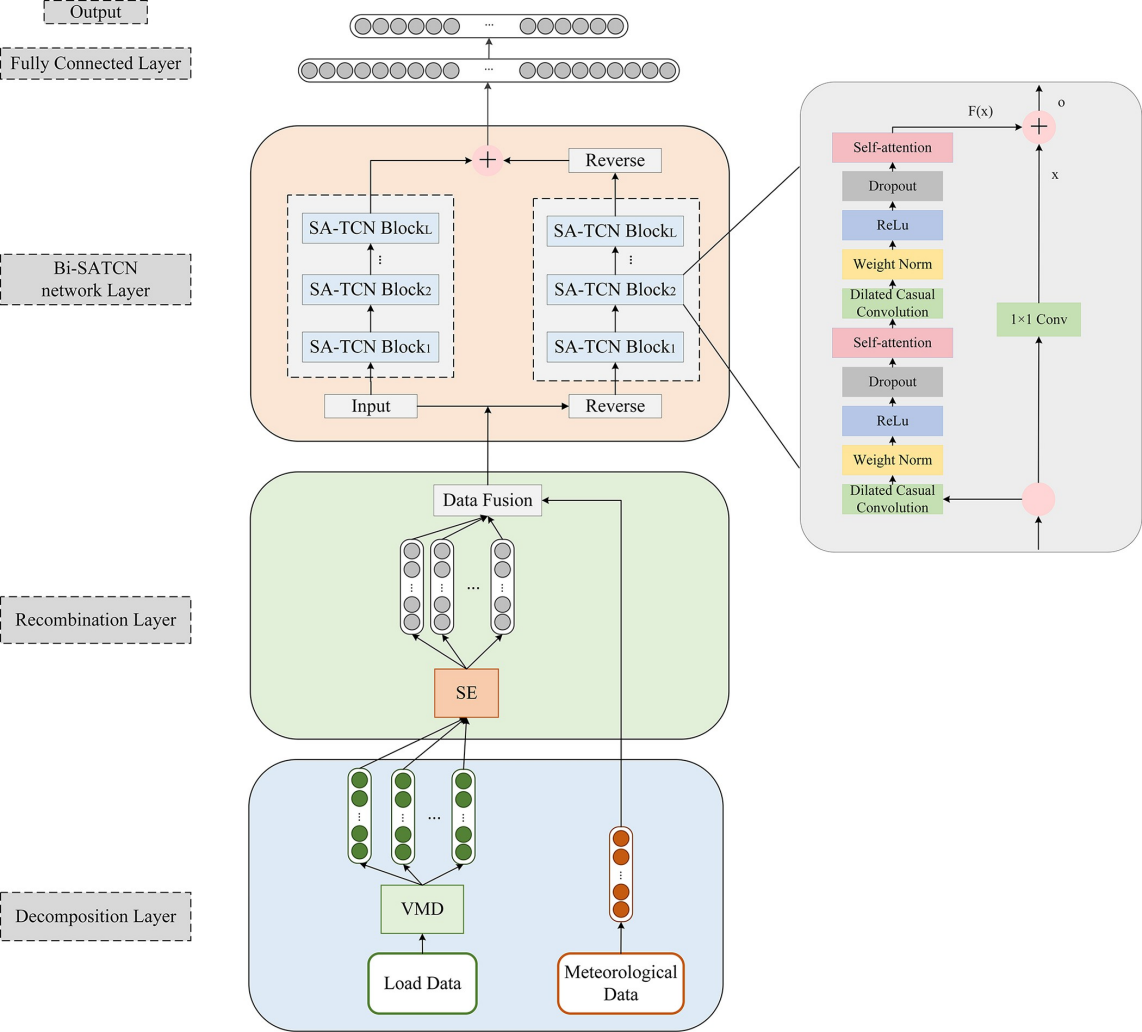
Power load forecasting framework diagram.

This research introduces a novel forecasting model incorporating VMD, SE, and the Bi-SATCN network to accurately predict short-term power loads. Firstly, VMD is utilized to decompose the highly unstable load sequence into multiple intrinsic mode functions (IMFs) with different center frequencies. The decomposed IMF component sequences are subsequently reconstructed using their sample entropy values, creating new component sequences termed SE-IMF. Each component is integrated with meteorological factors. The extracted features are then fed into the Bi-SATCN network for training and prediction. The Bi-SATCN network enhances the TCN model by incorporating a self-attention mechanism module into its framework. This modification effectively captures long-term dependencies and critical information. Additionally, to address the issue of insufficient forward and backward data correlation, a bidirectional TCN model structure was developed. Fig 5 illustrates the basic structure of the prediction model.

The model consists of three primary components: a time series decomposition layer, a reconstruction layer for decomposition sequences based on sample entropy, and a Bi-SATCN neural network prediction layer.

### 3.1 Time series decomposition layer

**Fig 6:**
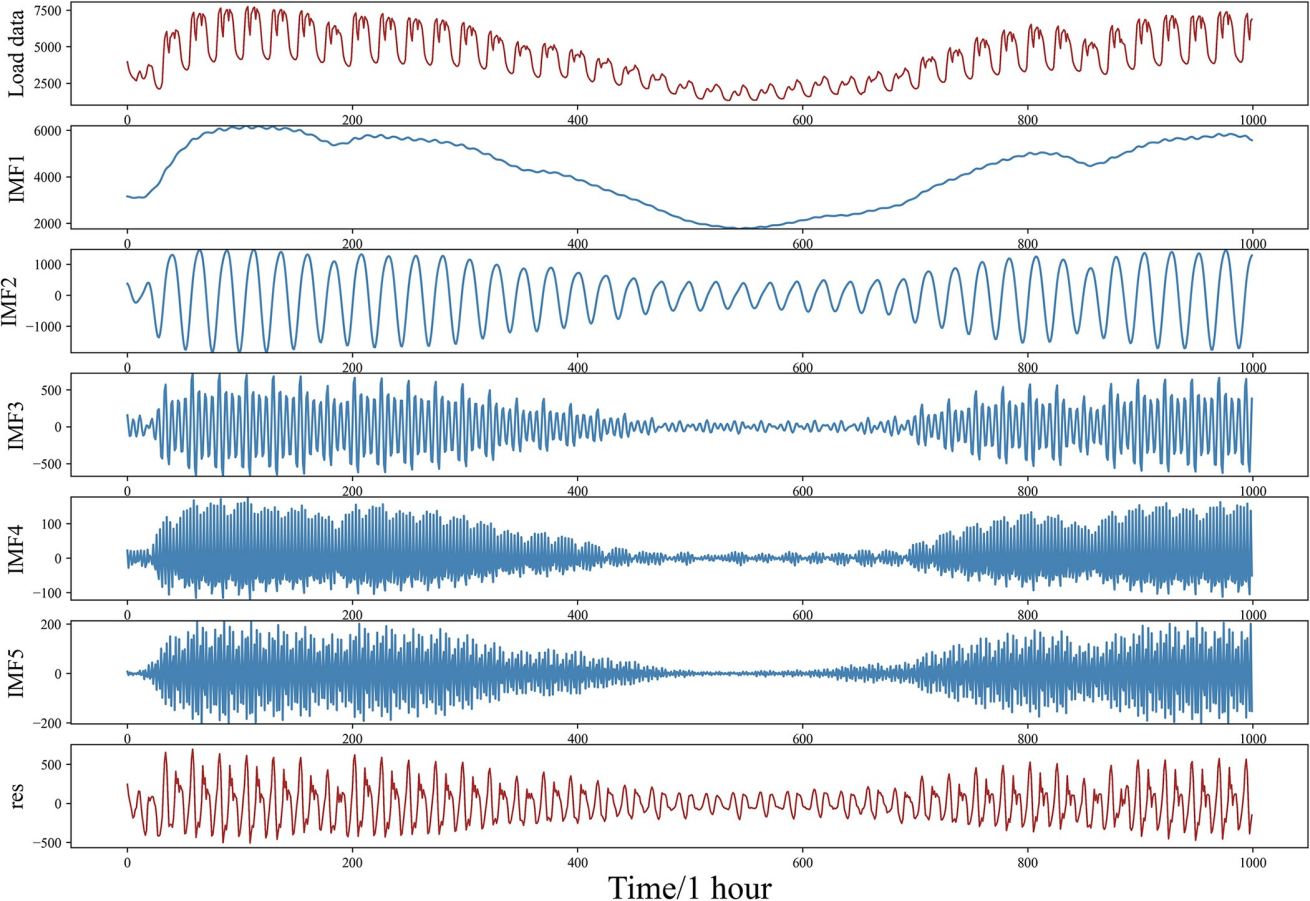
VMD decomposition results.

The raw data consists of daily meteorological data *m*(*t*) gathered daily and hourly load data *f*(*t*). In the experiment, the load data *f*(*t*) was decomposed using VMD to obtain five IMF components that are relatively smooth. Fig 6 illustrates the outcome of this decomposition.

By observing Fig 6, from top to bottom, the original, undecomposed power load data is shown, followed by the five IMF components derived from the decomposition. Each IMF sequence exhibits certain similarities. IMF2, IMF3, IMF4, and IMF5 all oscillate around the 0-axis and exhibit symmetry around the 0-axis. However, IMF4 displays more tightly spaced oscillations. The fluctuations of IMF1 and IMF2 are comparatively infrequent, with IMF1 showing the smallest amplitude. The trend of IMF1 aligns with the general trend of the original load.

### 3.2 Decomposition sequence reconstruction layer based on sample entropy

**Fig 7:**
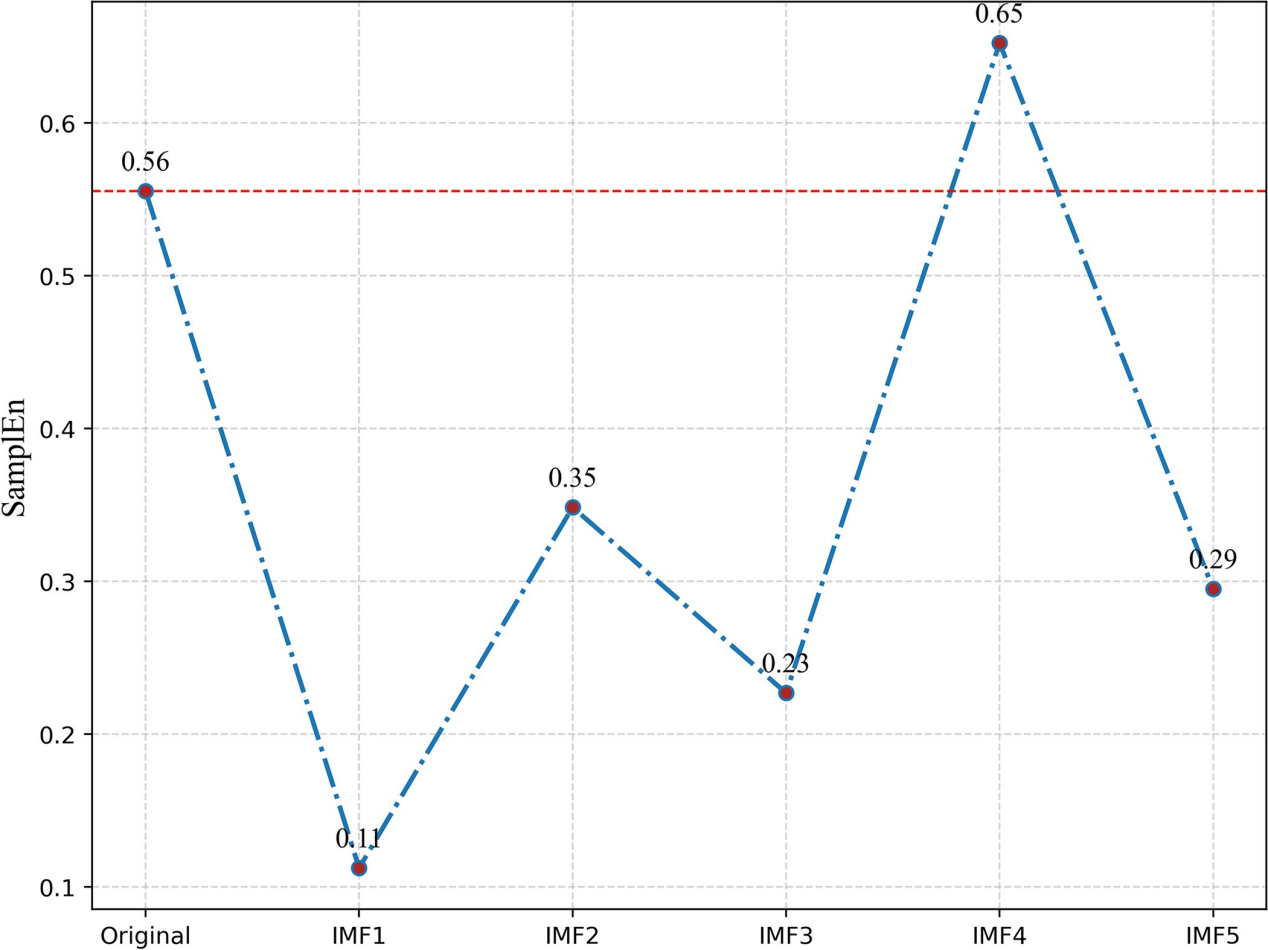
Sample entropy value of IMF.

Reconstructing highly similar IMFs is beneficial for enhancing the efficiency of load forecasting and decreasing the computational workload. In this subsection, sample entropy (SE) is used to calculate the complexity of each IMF derived from the decomposition in the preceding section. IMF components with similar SE values are then combined and reorganized into new components (SE-IMF). The similarity tolerance *r* is set to 0.1Std, and the parameter dimension *m* is set to 2, as introduced in Section 2.2. Fig 7 illustrates the calculation results of sample entropy.

**Fig 8:**
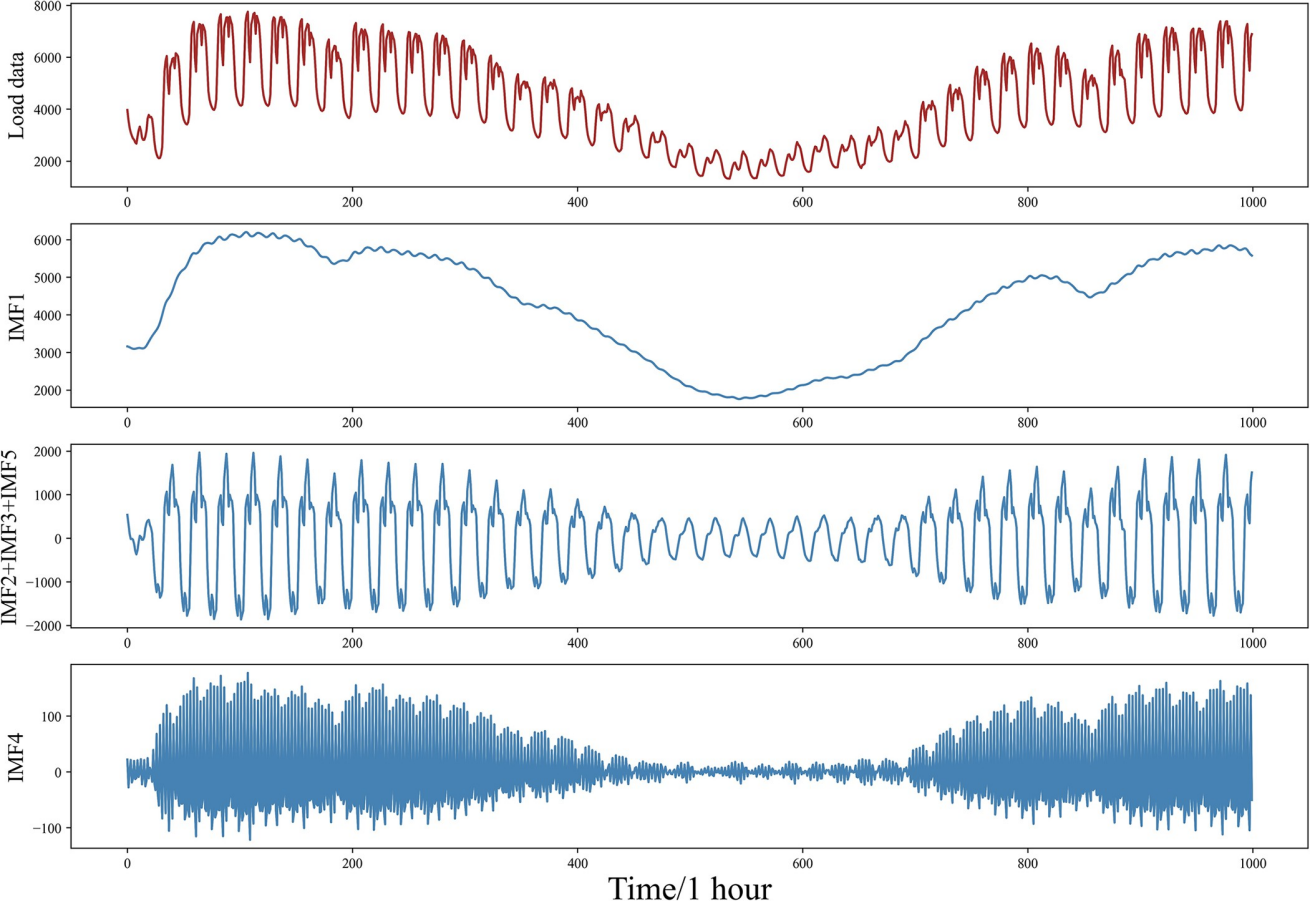
Reconstructed sequence.

The sample entropy value reflects the complexity or irregularity within the signal. Higher sample entropy values indicate a more complex signal, whereas lower sample entropy values suggest a more regular signal. It can be observed that the sample entropy value of the original load sequence is 0.56, and the sample entropy values for IMF1—IMF5 are 0.11, 0.35, 0.23, 0.65, and 0.29, respectively. Since the sample entropy value of IMF4 is higher than that of the original sequence, it is classified as a new high-frequency sequence; IMF2, IMF3, and IMF5 are grouped into a mid-frequency sequence, and IMF1 is designated as a low-frequency sequence. Fig 8 illustrates the outcome of the recombination.

In Fig 8, IMF1 is renamed as the low-frequency sequence SE-IMF1, while IMF2, IMF3, and IMF5 are merged and renamed SE-IMF2. IMF4, which exhibits the most tightly fluctuating behavior, is named the fluctuation sequence SE-IMF3.

### 3.3 Bi-SATCN neural network prediction layer

**Fig 9:**
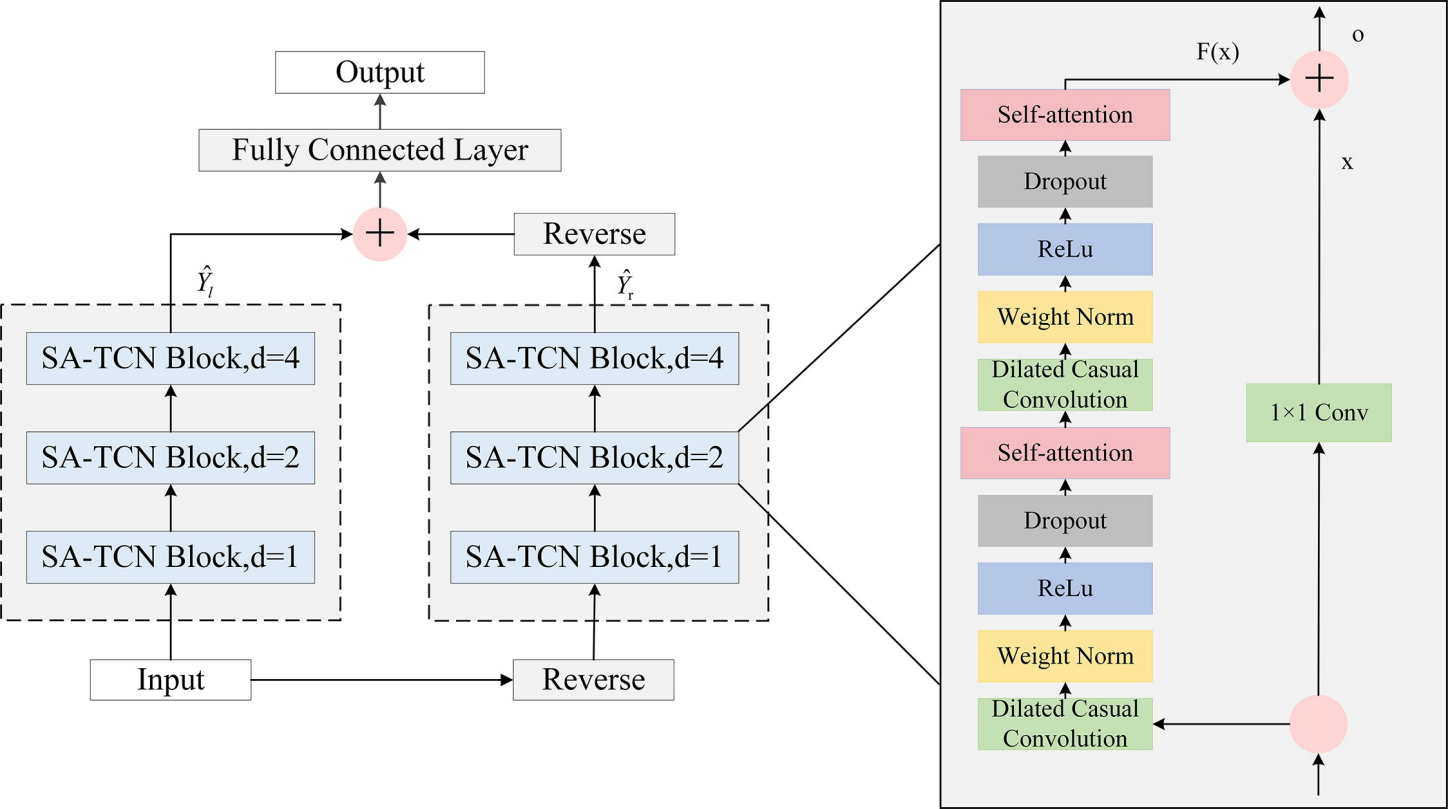
The architecture of Bi-SATCN.

This research will utilize the Bi-SATCN network model to forecast power load. By incorporating self-attention into the TCN network architecture, it is possible to emphasize important features in the data that are crucial for load prediction. This is achieved by assigning different weights to emphasize and capture the internal structural features of the sequence. Furthermore, the Bi-SATCN network takes into account the correlation between data nodes in both forward and backward directions by utilizing bidirectional hidden layers in its learning mechanism. Fig 9 illustrates the architecture of the Bi-SATCN neural network.

As illustrated in Fig 9, the Bi-SATCN architecture consists of forward SATCN and backward SATCN. Let *X* denote the load sequence, where *N* is the length of *X*, *X* = {*x*_1_,*x*_2_,…,*x*_*N*_}, and $\overset{\leftarrow }{X}=\{x_{N},x_{N-1},\ldots ,x_{1}\}$ represents the reverse sequence of *X*. By feeding the forward sequence *X* into the forward SATCN, the output is denoted by $\hat{Y_{l}}$, Similarly, the reverse sequence $\overset{\leftarrow }{X}$ is fed into the backward SATCN, with the output is denoted by $\hat{Y_{r}}$. The Bi-SATCN can be expressed as follows:

$$\hat{Y_{l}}=SATCN(X)$$ (12)

$$\hat{Y_{r}}=\overset{\leftarrow }{SATCN}(\overset{\leftarrow }{X})$$ (13)

$$Output=\hat{Y_{l}}⊕\overset{\leftarrow }{\hat{Y_{r}}}$$ (14)

where SATCN denotes the forward SATCN, $\overset{\leftarrow }{SATCN}$ denotes the backward SATCN, ⊕ represents the matrix addition operation, $\overset{\leftarrow }{\hat{Y_{r}}}$ is the inverse of $\hat{Y_{r}}$, and Output represents the final output of Bi-SATCN.

The network output at the present time $t,\hat{Y_{t}}$, is given by:

$$\hat{Y_{t}}=SATCN(x_{1},x_{2},\ldots ,x_{t})⊕\overset{\leftarrow }{SATCN}(x_{N},x_{N-1},\ldots ,x_{t})$$ (15)

In the network, SATCN operates on inputs from time *t* andearlier (*x*_1_,*x*_2_,…,*x*_t_), and $\overset{\leftarrow }{SATCN}$ operates on inputs from time *t* and later (*x*_*N*_,*x*_*N*−1_,…,*x*_*t*_). This bidirectional approach enables the network to leverage deep interactions, fully extracting features through both forward and backward processing.

### 3.4 Forecasting model process

**Fig 10:**
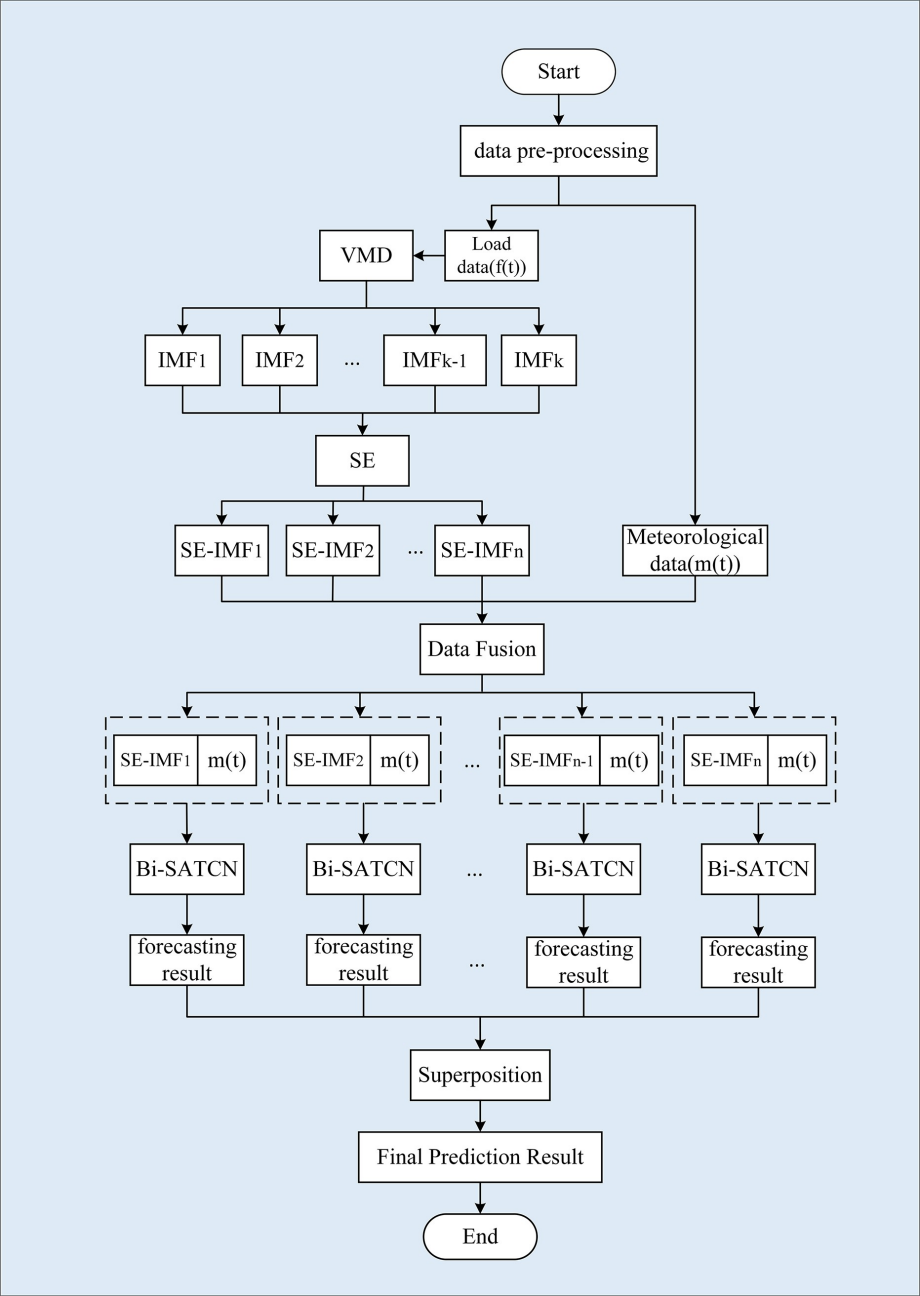
Flowchart of the Bi-SATCN model.

Fig 10 illustrates the flowchart for short-term power load forecasting using the proposed model. The steps involved in the procedure are as follows:

Step 1: Divide the data into load data *f*(*t*) and meteorological data *m*(*t*). Load forecasting results are influenced by various external factors, with changes in meteorological conditions having a direct and immediate impact on power loads. The most commonly considered meteorological data include temperature, humidity, rainfall, and other related factors.

Step 2: Using VMD, the highly fluctuating load data is decomposed into several IMF components of different frequencies.

Step 3: After calculating each IMF’s sample entropy values, new subsequences (SE-IMF) are created by recombining components with similar complexity.

Step 4: Each of the reconstructed components is individually fused with the meteorological data *m*(*t*).

Step 5: The fused data is fed into the Bi-SATCN network for training. The model’s hyperparameters are updated and modified by computing the loss function MSE.

Step 6: The Bi-SATCN network is used to make predictions for each subsequence. These predictions are then generated by the fully connected layer.

Step 7: The final forecast is obtained by aggregating the predicted values of the components. The assessment indicators used to evaluate the model’s predictive power include Mean Absolute Percentage Error (MAPE), Coefficient of Determination (R^2^), Mean Absolute Error (MAE), and Root Mean Square Error (RMSE).

## 4. Experimental analysis

### 4.1 Experimental setting and data analysis

Using the PyCharm Community Edition 2023.2 x64 development environment, the model was developed for the experiments using the TensorFlow framework.

**Fig 11:**
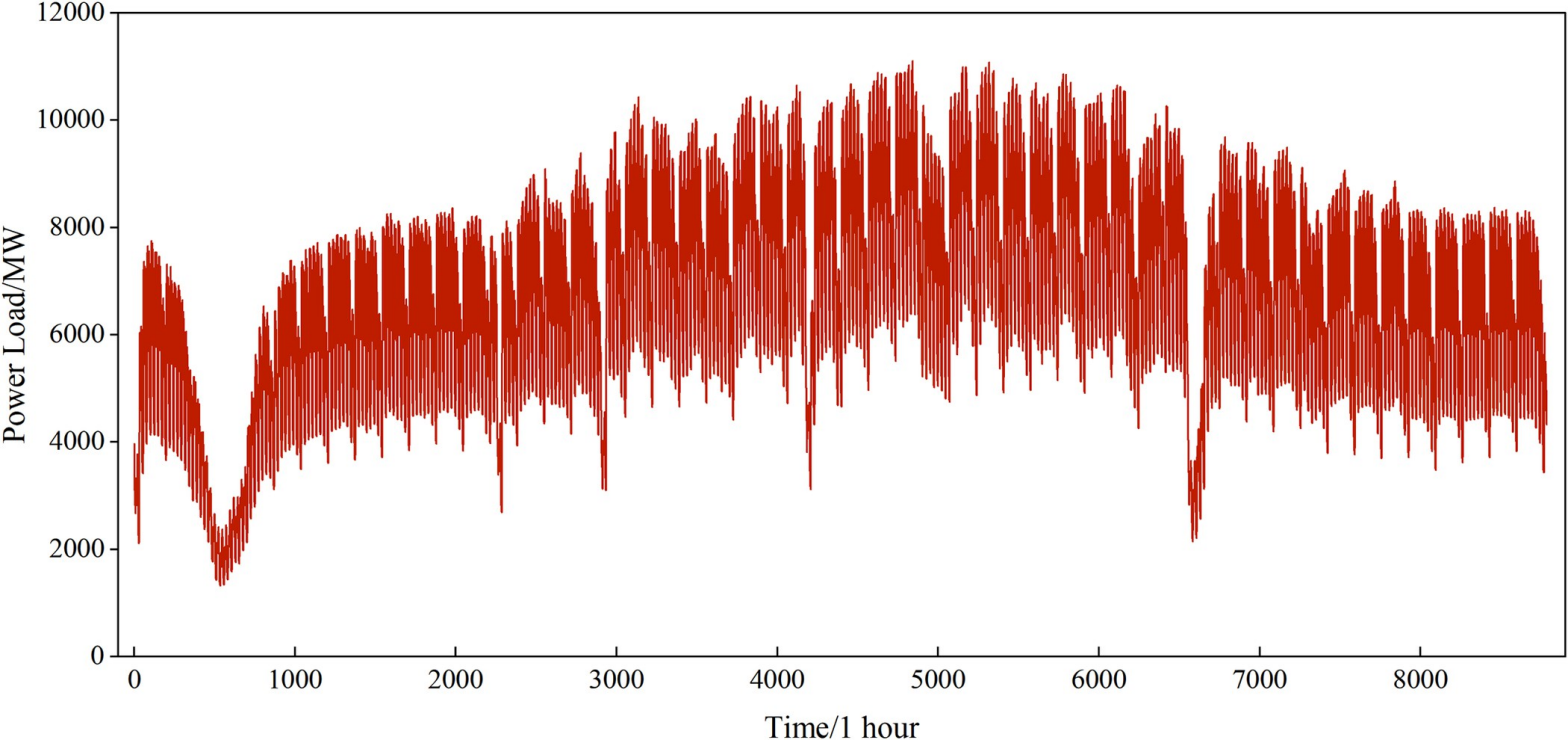
Hourly power load data from original dataset.

The power load dataset from Region 1 of the 9th China Electrical Mathematical Modeling Competition was used as the experimental data in this study. The dataset includes power load data and meteorological data from January 1, 2012, to December 31, 2012. The power load data was sampled at 1-hour intervals, with 24 time points per day, resulting in a total of 8,760 time points, as shown in Fig 11. The meteorological factors include the daily maximum temperature, daily minimum temperature, average temperature, relative humidity, and rainfall. Table 2 presents a portion of the meteorological data details (from January 1, 2012, to January 7, 2012). Additionally, real power load data from Shijiazhuang City, Hebei Province, was selected to validate the applicability and scalability of the proposed model. This data includes power load data and daily meteorological data from July 1, 2021, to April 26, 2022, also sampled at 1-hour intervals, with 8,760 time points in total. All datasets were divided, with the first 356 days used as the training set and the last 10 days as the test set, to evaluate the model’s performance.

**Table 2:** Meteorological data part details on the public dataset.

Date	Max. Temperature (°C)	Min. Temperature (°C)	Avg. Temperature (°C)	Relative Humidity(avg.)	Rainfall(mm)
1 January 2012	19.5	12.1	15.8	63	0
2 January 2012 3 January 2012 … 29 December 2012 30 December 2012 31 December 2012	20 18.7 … 22.3 11.9 12.5	13 14.2 … 10.2 6.8 4.1	16 15.8 … 18.3 8.8 8.3	59 72 … 80 61 28	0 0 … 0.4 3 0

### 4.2 Evaluation indicators

The experiments used root mean square error (RMSE), mean absolute percentage error (MAPE), coefficient of determination (R^2^), and absolute error (MAE) as evaluation indexes. Smaller values of MAE, MAPE, and RMSE indicate higher accuracy. Similarly, the closer the R^2^ value is to 1, the higher the prediction accuracy. The definitions of these four evaluation indicators are given by Eqs (16) to (20).

$$MAPE=\frac{1}{N}\frac{\sum_{i=1}^{N}\left|{\hat{Y}}_{i}-Y_{i}\right|}{Y_{i}}\times 100\%$$ (16)

$$RMSE=\sqrt{\frac{1}{N}\sum_{i=1}^{N}{\left({\hat{Y}}_{i}-Y_{i}\right)}^{2}}$$ (17)

$$MAE=\frac{1}{N}\sum_{i=1}^{N}\left|{\hat{Y}}_{i}-Y_{i}\right|$$ (18)

$$R^{2}=1-\frac{\sum_{i=1}^{N}{\left({\hat{Y}}_{i}-\bar{Y}\right)}^{2}}{\sum_{i=1}^{N}{\left(Y_{i}-\bar{Y}\right)}^{2}}$$ (19)

$$\bar{Y}=\frac{1}{N}\sum_{i=1}^{N}Y_{i}$$ (20)

where *Y*_*i*_ and ${\hat{Y}}_{i}$ are the actual and predicted values of the load at that time, $\bar{Y}$ is defined in Eq (20), and *N* is the total number of load points in the forecast.

### 4.3 Selection of hyperparameter

The key parameters of the Bi-SATCN network include the size of the convolutional kernel, the number of network layers, and the dilation factor. For small-scale parameter optimization, the grid search method is particularly efficient and feasible. The GridSearchCV method in Python was used to loop through the key parameters for optimization. The parameters were optimized using the Adam optimizer, with the mean square error (MSE) selected as the loss function. Additionally, an early stopping mechanism based on MSE was integrated into the model framework, with the patience parameter set to 15 and the significance threshold set to 0.1%. If the MSE does not decrease by more than 0.1% over 15 consecutive training epochs, training is stopped as a measure to prevent overfitting. Table 3 details the parameter settings for this model on both the public and real datasets. The optimal model parameter configuration was determined through experimentation.

**Table 3:** Perparameters on the public dataset and real dataset.

Dataset	Parameter	Range	Result
public dataset	Number of Residual Blocks	(2,3,4,5)	3
	Convolution Kernel Size	(2,3,4)	3
	Dilation factor	(1,2,4,8,16,32)	(1,2,4)
	Batch Size	(16,32,64)	32
	learning rate	[0.00001,0.01]	0.001
	Dropout Epoch	(0.2,0.3,0.4) ——	0.2 100
real dataset	Number of Residual Blocks	(2,3,4,5)	5
	Convolution Kernel Size	(2,3,4)	2
	Dilation factor	(1,2,4,8,16,32)	(1,2,4,8,16)
	Batch Size	(16,32,64)	32
	learning rate	[0.00001,0.01]	0.001
	Dropout Epoch	(0.2,0.3,0.4) ——	0.2

### 4.4 Proposed method

**Fig 12:**
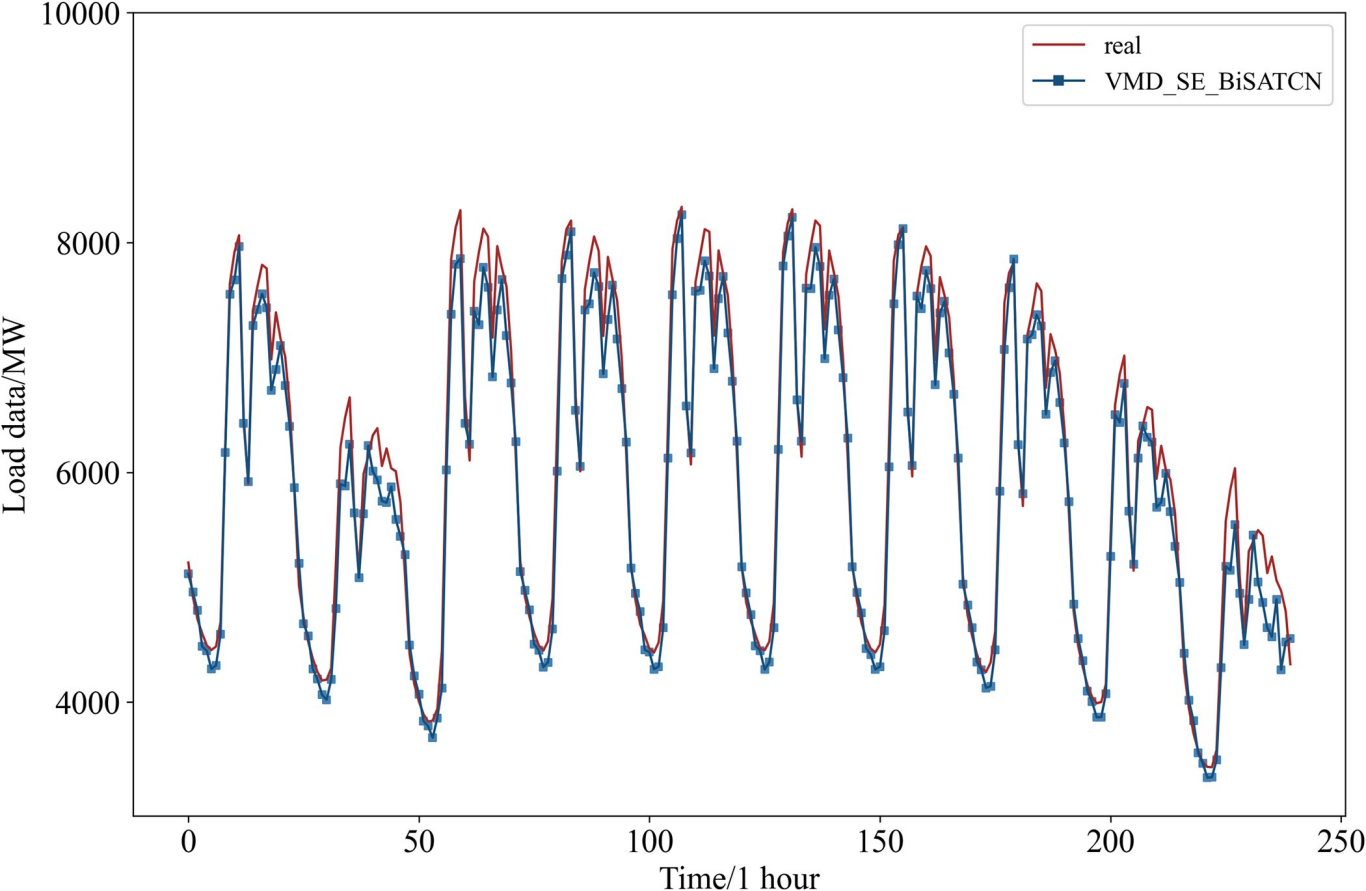
Prediction results for the public dataset.

The hyperparameters were set as indicated in Table 1. In addition to using public datasets, real datasets were also collected to validate the model’s effectiveness. The final predictions for the public dataset are illustrated in Fig 12.

In Table 4, the proposed method achieved a MAPE of 0.81%, RMSE of 126.9058 MW, MAE of 92.8696 MW, and an R^2^ of 99.18%.

**Table 4:** Evaluation metrics for public dataset.

Proposed Method	MAPE (%)	RMSE(MW)	MAE(MW)	*R* ^2^
VMD-SE-BiSATCN	0.81	126.9058	92.8696	0.9918

**Fig 13:**
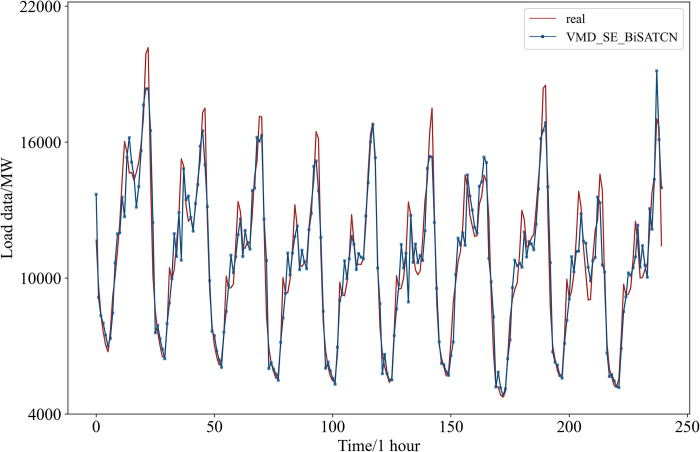
Prediction results for the real dataset.

The final prediction of the real data is shown in Fig 13. In Table 5, the proposed method achieved a MAPE of 3.19%, RMSE of 1043.1784 MW, MAE of 682.3129 MW, and an R^2^ of 90.48%. The comparative tests in the next section will provide further validation of the model’s predictive accuracy.

**Table 5:** Evaluation metrics for the real data.

Proposed Method	MAPE (%)	RMSE(MW)	MAE(MW)	*R* ^2^
VMD-SE-BiSATCN	3.19	1043.1784	682.3129	0.9048

### 4.5 Comparative experiment

#### 4.5.1 Ablation experiment

**Fig 14:**
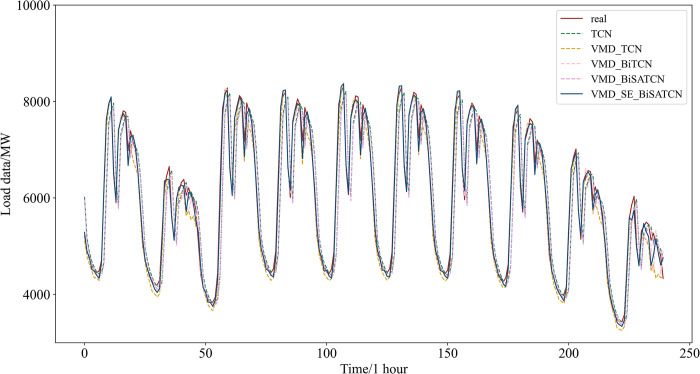
Comparison of predicted results from ablation experiments.

To evaluate the predictive efficacy of each stage of the model described in this study, four models were constructed: the TCN model, the TCN model based on VMD, the BiTCN model based on VMD, and the BiSATCN model based on VMD. These models were then compared with the final VMD-SE-BiSATCN model proposed in this study. Fig 14 illustrates the predictive performance of the aforementioned comparison models, and the prediction error evaluation metrics for each model are presented in Table 6.

**Table 6:** Evaluation metrics of the ablation experiment model.

TCN	+VMD	+Bi	+SA	+SE	MAPE (%)	RMSE(MW)	MAE(MW)	*R* ^2^
**√**					3.85	680.425	480.442	0.766
**√**	**√**				1.51**(-2.34)**	221.881**(-458.544)**	176.271**(-304.171)**	0.975**(+0.210)**
**√**	**√**	**√**			1.02**(-0.49)**	168.407**(-53.475)**	122.804**(-53.466)**	0.986**(+0.011)**
**√**	**√**	**√**	**√**		0.97**(-0.05)**	146.212**(-22.195)**	111.28 **(-11.521)**	0.989**(+0.004)**
**√**	**√**	**√**	**√**	**√**	0.81**(-0.16)**	126.906 **(-19.306)**	92.870 **(-18.413)**	0.992**(+0.003)**

Table 6 demonstrates that the VMD-SE-BiSATCN model, developed in this study, exhibits the lowest MAPE, RMSE, and MAE values of 0.81%, 126.9058 MW, and 92.8696 MW, respectively, when compared to other models. Additionally, it achieves the highest R^2^ value of 0.9918. As the most fundamental model, the TCN model exhibits comparatively inferior prediction outcomes, thereby demonstrating the efficacy of time series decomposition when compared to the VMD-TCN model. When comparing the remaining three models based on VMD, it is evident that the prediction accuracy of the VMD-BiSATCN model is superior. This highlights the positive impact of incorporating the self-attention mechanism and constructing a bidirectional model, leading to improved prediction accuracy. Ultimately, the accuracy of the VMD-SE-BiSATCN model surpasses that of the VMD-BiSATCN model, indicating the usefulness of sample entropy-based time series reconstruction. In conclusion, the VMD-SE-BiSATCN model developed in this study yields the most accurate prediction results.

#### 4.5.2 Comparison and analysis of different models

Experiments on an identical dataset are conducted to verify the effectiveness of the methodology proposed in this study. The evaluation metrics of various models are derived by comparing them against SVR, LSTM, Bi-LSTM, and TCN-LSTM models. The results of these metrics are then analyzed.

The following are the comparison models.

1. **SVR model** [41]: A traditional forecasting model based on regression analysis.
2. **LSTM model** [19]: This model effectively addresses the issues of gradient explosion and vanishing that occur in conventional recurrent neural networks (RNNs) when handling long-term sequences.
3. **Bi-LSTM model** [20]: A bidirectional neural network based on LSTM that performs both forward and backward computation.
4. **TCN-LSTM model** [42]: Combines the TCN and LSTM models to extract data features using TCN’s convolutional operations and predict time series changes using the LSTM model.
5. **VMD-SE-BiSATCN model**: The model proposed in this study.

**Fig 15:**
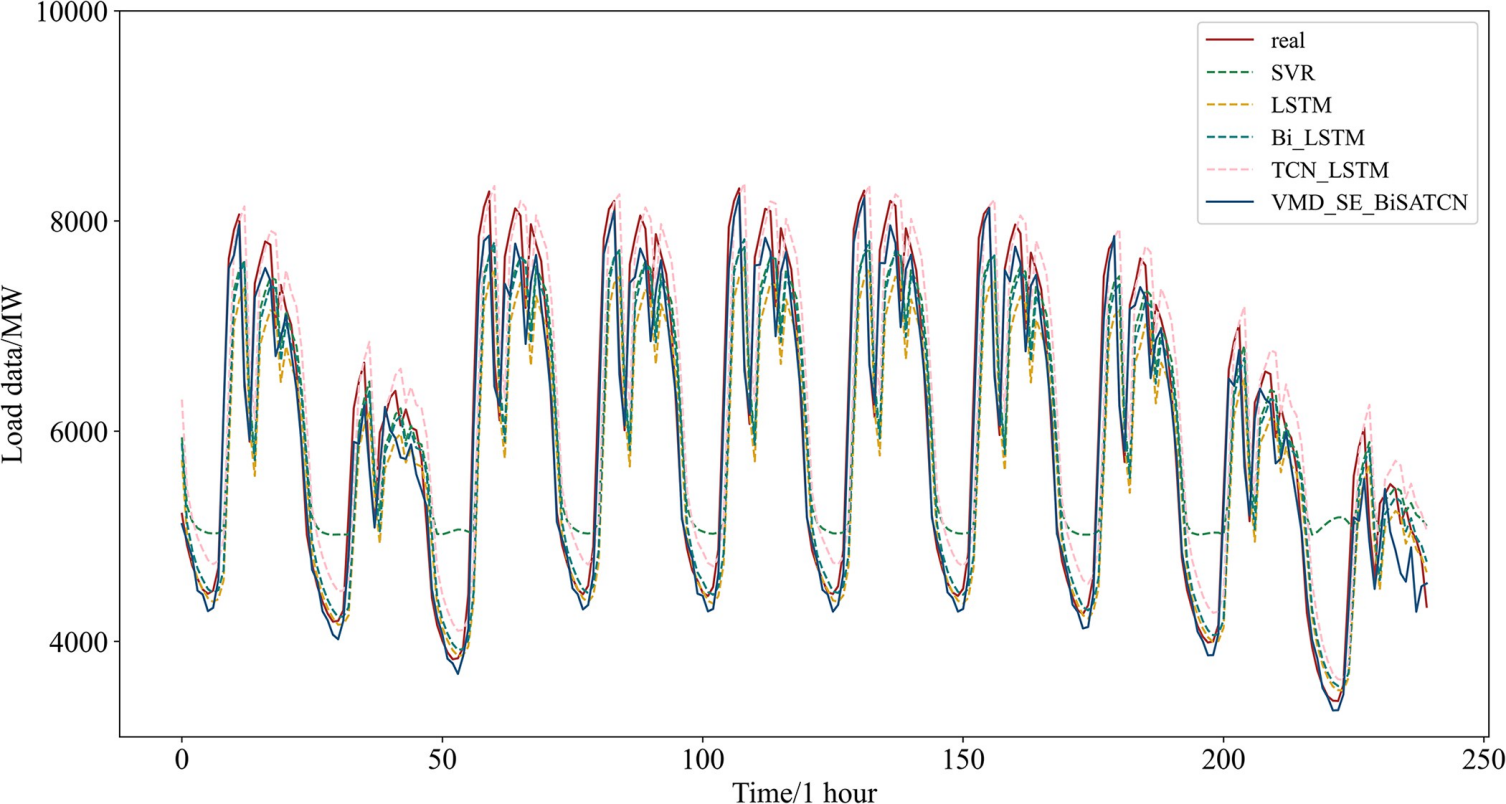
Comparative experimental prediction results for the public dataset.

Fig 15 shows the predicted outcomes of each model. The VMD-SE-BiSATCN model predicts results that are closer to the power loads in the public dataset. The evaluation indicators for each of the prediction models are presented in Table 7. The table demonstrates that our proposed model outperforms the other four experimental models. Specifically, the model achieved a MAPE of 0.81%, RMSE of 126.9058 MW, and MAE of 92.8696 MW. The MAPE, the primary evaluation index, was reduced by 85.9%, 79.7%, 78.9%, and 78.7%, respectively. In addition, the R^2^ for the proposed model is 0.9918, indicating a strong correlation with the actual data.

**Table 7:** Evaluation metrics for comparative experiments with the public dataset.

Model	MAPE (%)	RMSE(MW)	MAE(MW)	*R* ^2^
SVR	5.73	763.6242	637.7833	0.7046
LSTM	3.99	695.7752	490.1236	0.7548
Bi-LSTM	3.84	687.407	488.2705	0.7606
TCN-LSTM	3.81	664.9761	480.935	0.776
VMD-SE-BiSATCN	0.81	126.9058	92.8696	0.9918

## 5. Conclusion

This study proposes a Bi-SATCN network for short-term power load forecasting, integrating VMD and SE. The SE technique is utilized to evaluate the sequences decomposed by each VMD. The components with similar features are subsequently recombined and fed into the forecasting model. Furthermore, the forecasting model utilizes the Bi-SATCN neural network, which incorporates the self-attention mechanism and establishes a bidirectional model structure. The final load prediction is obtained by summing the predicted outcomes from each component. This approach effectively improves the prediction accuracy for highly volatile and nonlinear load series.

To assess the efficacy of the VMD-SE-BiSATCN model, four comparable forecasting models were also constructed. The outcomes of the forecasting analysis demonstrate that the proposed VMD-SE-BiSATCN method outperforms the other four models in short-term power load prediction.

For future research, we propose incorporating additional influencing factors, such as market characteristics, population density, and the level of economic development. Furthermore, this technique could be applied to make predictions in other fields, such as forecasting solar power generation and air quality.

## Supporting information

S1 File Experimental data. (ZIP)
